# Suppression of LPS-induced matrix-metalloproteinase responses in macrophages exposed to phenytoin and its metabolite, 5-(p-hydroxyphenyl-), 5-phenylhydantoin

**DOI:** 10.1186/1476-9255-7-48

**Published:** 2010-09-15

**Authors:** Ryan Serra, Abdel-ghany Al-saidi, Nikola Angelov, Salvador Nares

**Affiliations:** 1 Department of Periodontology, School of Dentistry, University of North Carolina at Chapel Hill, Chapel Hill, North Carolina, USA; 2 Department of Periodontics, School of Dentistry, Loma Linda University, Loma Linda, CA, 92350, USA

## Abstract

**Background:**

Phenytoin (PHT) has been reported to induce gingival (gum) overgrowth (GO) in approximately 50% of patients taking this medication. While most studies have focused on the effects of PHT on the fibroblast in the pathophysiology underlying GO, few studies have investigated the potential regulatory role of macrophages in extracellular matrix (ECM) turnover and secretion of proinflammatory mediators. The aim of this study was to evaluate the effects of PHT and its metabolite, 5-(p-hydroxyphenyl-), 5-phenylhydantoin (HPPH) on LPS-elicited MMP, TIMP, TNF-α and IL-6 levels in macrophages.

**Methods:**

Human primary monocyte-derived macrophages (*n *= 6 independent donors) were pretreated with 15-50 μg/mL PHT-Na^+ ^or 15-50 μg/mL HPPH for 1 hour. Cells were then challenged with 100 ng/ml purified LPS from the periodontal pathogen, *Aggregatibacter actinomycetemcomitans*. Supernatants were collected after 24 hours and levels of MMP-1, MMP-2, MMP-3, MMP-9, MMP-12, TIMP-1, TIMP-2, TIMP-3, TIMP-4, TNF-α and IL-6 determined by multiplex analysis or enzyme-linked immunoadsorbent assay.

**Results:**

A dose-dependent inhibition of MMP-1, MMP-3, MMP-9, TIMP-1 but not MMP-2 was noted in culture supernatants pretreated with PHT or HPPH prior to LPS challenge. MMP-12, TIMP-2, TIMP-3 and TIMP-2 were not detected in culture supernatants. High concentrations of PHT but not HPPH, blunted LPS-induced TNF-α production although neither significantly affected IL-6 levels.

**Conclusion:**

The ability of macrophages to mediate turnover of ECM via the production of metalloproteinases is compromised not only by PHT, but its metabolite, HPPH in a dose-dependent fashion. Further, the preferential dysregulation of macrophage-derived TNF-α but not IL-6 in response to bacterial challenge may provide an inflammatory environment facilitating collagen accumulation without the counteracting production of MMPs.

## Background

Drug-induced gingival (gum) overgrowth (DIGO) is widely recognized as a common unwanted sequelae associated with a variety of medications. Among these, the antiepileptic agent, PHT (Dilantin^®^), has been reported to induce gingival overgrowth (GO) in approximately 50% of patients taking this medication [1,2]. PHT is a hydantoin-derivative anticonvulsant that exerts its anticonvulsant properties by stabilizing neuronal cell membranes to the action of sodium, potassium, and calcium. The drug also affects the transport of calcium across cell membranes and decreases the influx of calcium ions across membranes by decreasing membrane permeability and blocking intracellular uptake [3]. PHT is primarily metabolized by liver cytochrome P450 enzymes, particularly CYP2C9 and CYP2C19 [4] to form enantiomers of 5-(4-hydroxyphenyl-),5-phenylhydantoin (HPPH) which in addition to PHT, have been implicated in the pathogenesis of DIGO [5,6].

While most studies have focused on the role of the fibroblast [7-10], it is likely that other cells contribute to the pathogenesis of DIGO. In particular, tissue macrophages, present in elevated numbers within gingival tissues, possibly in response to accumulation of the plaque biofilm [2,11], may play a role in pathogenesis. These long-lived, multifaceted cells, strategically poised along portals of entry, perform numerous functions of vital importance to the host. In addition to their key role in immunity [12], the macrophage is recognized as the major mediator of normal connective tissue turnover and maintenance, as well as for orchestrating repair during wound healing [13-18]. It has a dualistic role to receive, amplify, and transmit signals to fibroblasts, endothelial cells, and vascular smooth muscle cells by producing pro-inflammatory and catabolic cytokines. However, during tissue turnover and wound healing it secretes anabolic peptide growth factors [12]. Given this duality of function, any perturbation can lead to pathological processes. We have demonstrated that the clinical presentation of PHT-induced gingival overgrowth is associated with a specific macrophage phenotype characterized by high expression levels of IL-1β and PDGF-B [11,19] suggesting that this drug-induced macrophage phenotype could contribute to the pathogenesis of DIGO. These cellular attributes might explain the dichotomy of the lesion where there is both periodontal inflammation typically associated with connective tissue catabolism paradoxically juxtaposed with gingival overgrowth,- a clear anabolic signal of wound repair and regeneration.

As tissue homeostasis requires the proper balance of metabolism and catabolism, it is possible that macrophage-derived cytokines, MMPs and TIMP levels are altered in response to PHT and HPPH. Here we investigated the effects of these agents on production of MMPs, TIMPs, and pro-inflammatory cytokines in human monocyte-derived macrophages and report that indeed, PHT and HPPH significantly modulate macrophage MMP and cytokine protein levels in response to purified LPS from the periodontal pathogen, *Aggregatibacter actinomycetemcomitans*.

## Methods

### Monocyte isolation and macrophage differentiation

Peripheral blood mononuclear cells were obtained from commercially-available buffy coats (Oklahoma Blood Institute, Oklahoma City, OK, USA) derived from healthy donors by density gradient centrifugation using Ficoll-paque (Amersham, Uppsala, Sweden). Six independent cultures were obtained from 6 independent donors. Monocytes were isolated using CD14 MicroBeads (Miltenyi Biotec, Auburn, CA, USA) according to manufacturer's instructions and cultured as previously described [12,20,21]. Briefly, isolated monocytes were plated onto duplicate 12-well tissue culture-treated plates (BD Biosciences, San Jose, CA, USA) at a density of 5 × 10^5 ^cells/cm^2 ^in serum-free DMEM with L-glutamine (Cellgro, Manassas, VA, USA) containing 50 μg/mL gentamicin (Sigma, St. Louis, MO, USA) at 37 C, 5% CO_2 _to promote monocyte attachment. After 2 hours, heat-inactivated fetal bovine serum (FBS, Invitrogen, Carlsbad, CA, USA) was added to a final concentration of 10%. Cells were >95% CD14+ as determined by FACS analysis (data not shown) prior to culture.

### Macrophage stimulation

After 5 days, the media and non-adhered cells were removed and replaced with complete media (DMEM, 10% FBS, gentamicin) and incubated at 37 C, 5% CO_2_. Media was replaced every 2 days. Experiments were initiated upon confirmation of macrophage differentiation after 7 days in culture [12,20,21]. Macrophages were used between day 7 and 10 and pretreated with either: 1) 15 μg/mL of PHT-Na+ (Sigma), (serum levels, [22-24]), 2) 50 μg/mL PHT-Na+ (high dose), 3) 15 μg/mL PHT metabolite (Sigma), (5-(4'-hydroxyphenyl),5-phenylhydantoin, HPPH), or 4) 50 μg/mL HPPH for 1 hour. Untreated cells served as control cultures. Stock solutions of PHT-Na+ (150 mg/mL) were made in sterile deionized water while HPPH (150 mg/mL) solutions were made in DMSO. Each stock solution was further diluted prior to use. The total concentration of DMSO in cultures was always less than 0.05%. DMSO concentrations less than 0.1% have been reported not to affect cellular viability and function [25,26]. Nevertheless, we confirmed these findings in preliminary studies exposing macrophage cultures to 0.05% DMSO (data not shown). To induce production of MMPs and proinflammatory cytokines, macrophages were challenged with 100 ng/mL purified LPS from the Gram-negative, periodontal pathogen, *Aggregatibacter actinomycetemcomitans *(*A. actinomycetemcomitans *(Aa), serotype b, strain Y4, a kind gift from K. L. Kirkwood, University of South Carolina, USA) for 24 hours. Isolation and purification of Aa LPS has been previously described [27]. Previous studies have demonstrated that LPS from this organism is capable of inducing MMP and TIMP production [28-30] and our preliminary studies determined that this concentration of LPS was capable of significantly inducing TNF-α levels in human primary macrophages and THP-1 cells induced for macrophage differentiation (data not shown).

### MMP, TIMP protein assays

After 24 hours, the media was collected, spun at 12,000 × g, transferred to fresh tubes and stored at -80 C until further use. Quantification of supernatant MMP and TIMP levels were determined using the Luminex 100 System (Luminex Co., Austin, TX, USA) and the Fluorokine MAP Multiplex Human MMP Panel and the Fluorokine MAP Human TIMP Multiplex Kit, respectively according to the manufacturer's instructions (both from R & D, Minneapolis, MN, USA). These kits measure levels of pro-, mature, and TIMP complexed MMPs. Six independent experiments were performed from cells derived from 6 different donors. The assays were performed in 96-well plates, as previously described [20]. For MMP determination, microsphere beads coated with monoclonal antibodies against MMP-1, MMP-2, MMP-3, MMP-9, MMP-12 were added to the wells. For TIMP determination, microsphere beads coated with monoclonal antibodies against TIMP-1, TIMP-2, TIMP-3, and TIMP-4 were added to the wells of a separate plate. To remain below the upper level of quantitation, samples containing LPS were diluted 10-fold prior to analysis. This dilution factor was based on our preliminary studies. Samples and standards were pipetted into wells, incubated for 2 hours with the beads then washed using a vacuum manifold (Millipore Corporation, Billerica, MA USA). Biotinylated secondary antibodies were added and incubation for 1 h. The beads were then washed and incubated for an additional 30 minutes with streptavidin conjugated to the fluorescent protein, R-phycoerythrin (streptavidin/R-phycoerythrin). The beads were washed and analyzed (a minimum of 50 per analyte) using the Luminex 100 system. The Luminex 100 measures the amount of fluorescence associated with R-phycoerythrin, reported as median fluorescence intensity of each spectral-specific bead allowing it to distinguish the different analytes in each well. The concentrations of the unknown samples (antigens in macrophage supernatants) were estimated from the standard curve using a third-order polynomial equation and expressed as pg/mL after adjusting for the dilution factor. Samples below the detection limit of the assay were recorded as zero. The minimum detectable concentrations for the assays were as follows: MMP-1: 4.4 pg/mL, MMP-2: 25.4 pg/mL, MMP-3: 1.3 pg/mL, MMP-9: 7.4 pg/mL, TIMP-1: 1.54 pg/mL, TIMP-2: 14.7 pg/mL, TIMP-3: 86 pg/mL and TIMP-4: 1.29 pg/mL. All values were standardized for total protein using the Bradford assay (Pierce, Thermo Scientific, Rockford, IL, USA) according to manufacturer's instructions. Briefly, culture supernatants were mixed with assay reagent and incubated for 10 minutes at room temperature in 96 well plates. Bovine serum albumin (BSA, Invitrogen) was used as a standard. The absorbance at 595 nm was read using a SpectraMax M2 microplate reader (Molecular Devices, Sunnyvale, CA, USA). Values obtained from untreated control cultures were arbitrarily used as a baseline measure. The ratio, (control)/(supernatant protein value) was used to normalize each sample based on total protein.

### Cytokine assays

After 24 hours, supernatants (*n *= 6 independent donors) were collected and levels of TNF-α and IL-6 determined by ELISA (RayBiotech, Norcross, GA, USA) according to manufacturer's instructions. The absorbance at 450 nm was read using a SpectraMax M2 microplate reader (Molecular Devices) with the wavelength correction set at 550 nm. The rated sensitivities of the commercial ELISA kits was 15 pg/mL for TNF-α and 6 pg/mL for IL-6. Values were standardized for total protein using the Bradford assay as described above.

### Cell viability assays

Viability of macrophages was evaluated using the CellTiter 96 AQueous One Solution Cell Proliferation Assay [3-(4,5-diethylthiazol-2-yl)-5-(3-carboxymethoxyphenyl)-2-(4-sulfophenyl)-2H-tetrazolium, inner salt, MTS] assay according to the manufacturer's protocol (Promega, Madison, WI, USA). This colorimetric method can be used to determine the number of viable cells in proliferation or to evaluate cytotoxicity. Briefly, macrophages were cultured in triplicate in 96-well plates and treated with PHT, HPPH and LPS as described above. Unstimulated cells served as control cultures. After 24 h, the cells were incubated with MTS for 2 h at 37 C, 5% CO_2_. The absorbance was read at 490 nm using a microplate reader.

### Statistical analysis

Data were analyzed using a hierarchical multiple regression approach relative to LPS, drug and dose. The first tier sought to establish the validity of the positive control, LPS vs the negative control group. The second tier of this analysis was aimed at determining whether PHT or HPPH have an effect on MMP, TIMP, TNF-α and IL-6 levels. Finally, the third tier sought to contrast dose and compare one drug with another. Data were expressed as mean ± SEM and compared using a two-tailed Student's *t *test for correlated samples (GraphPad Prism, GraphPad Software, La Jolla, CA, USA). Results were considered statistically significant at *p *< 0.05.

## Results

### PHT and HPPH inhibit LPS-induced supernatant levels of MMP-1, MMP-3, MMP-9, and TIMP-1 in a dose dependent manner

**Figure 1:**
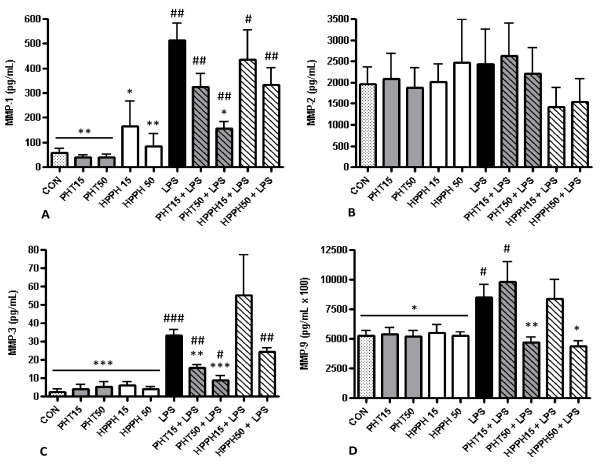
**The effect of phenytoin, HPPH and LPS on levels of (A) matrix metalloproteinase-1, (B) matrix metalloproteinase-2, (C) matrix metalloproteinase-3, and (D) matrix metalloproteinase-9 in conditioned medium from macrophage cultures**. Primary human monocyte-derived macrophages (n=6 independent cultures) were pretreated with phenytoin or HPPH (15 μg/mL and 50 μg/mL) for 1 hour prior to challenge with 100 ng/mL *A. actinomycetemcomitans* LPS and the levels of matrix metalloproteinase-1, matrix metalloproteinase-2, matrix metalloproteinase-3, and matrix metalloproteinase-9 measured after 24 hours in conditioned media by multiplex analysis. MMP-1, matrix metalloproteinase-1; MMP-2, matrix metalloproteinase-2; MMP-3, matrix metalloproteinase-3; MMP-9, matrix metalloproteinase-9; CON, control; PHT, phenytoin; HPPH, 5-(4-hydroxyphenyl-),5- phenylhydantoin; LPS, lipopolysaccharide. Compared to CON, # p<0.05, ## p<0.01, ### p<0.001, compared to LPS, * p<0.05, ** p<0.01, *** p<0.001. Student t-test, n=6 independent donors.

**Figure 2:**
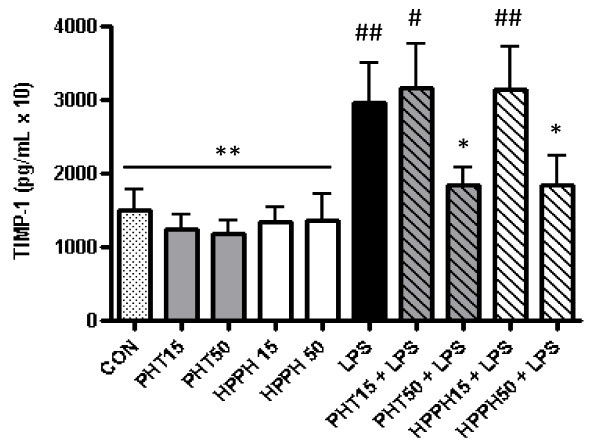
**The effect of phenytoin, HPPH and LPS on levels of tissue inhibitor of matrix metalloproteinase-1 in conditioned medium from macrophage cultures**. Primary human monocyte-derived macrophages (*n *= 6 independent cultures) were pretreated with phenytoin or HPPH (15 μg/mL and 50 μg/mL) for 1 hour prior to challenge with 100 ng/mL *A. actinomycetemcomitans *LPS and the levels of tissue inhibitor of matrix metalloproteinase-1 measured after 24 hours in conditioned media by multiplex analysis. TIMP-1, tissue inhibitor of matrix metalloproteinase-1; CON, control; PHT, phenytoin; HPPH, 5-(4-hydroxyphenyl-),5-phenylhydantoin; LPS, lipopolysaccharide. Compared to CON, # *p *< 0.05, ## *p *< 0.01, ### *p *< 0.001, compared to LPS, * *p *< 0.05, ** *p *< 0.01, *** *p *< 0.001. Student *t*-test, *n *= 6 independent donors.

To evaluate the effects of PHT and its metabolite, HPPH on macrophage MMP and TIMP levels, human monocyte-derived macrophages were pretreated for 1 hour with either 15 μg/mL or 50 μg/mL of these agents prior to challenge with LPS. Previous studies have determined that PHT plasma levels of 10-20 μg/mL are necessary to effectively maintain effective seizure control [22-24]. Thus, the concentrations used in our study represent therapeutic as well as elevated levels of PHT permitting the evaluation of dose on MMP and TIMP production. To rule out the possibility that differences in supernatant levels of these readouts were due to decreased cell viability, we performed a viability assay on cells cultured in each condition. No significant differences were noted in the viability of cells exposed to LPS and either dose of PHT, HPPH, PHT/LPS or HPPH/LPS as determined by MTS assay. Further, we standardized the results of each analyte to total protein concentration for each condition using a Bradford assay. No differences were noted for any analyte examined in conditioned media from macrophage cultures treated with PHT or HPPH alone compared to control cultures (*p *> 0.05). As expected, LPS markedly induced supernatant MMP-1, MMP-3, MMP-9, TIMP-1 but not MMP-2 levels in our 6 independent cultures after a 24 hour exposure (Fig. 1A-D). Compared to untreated control cultures, LPS significantly increased secretion of MMP-1 despite the presence of either PHT or HPPH at any dose. This was similarly observed for MMP-3 levels with the exception of cultures pretreated with 15 μg/mL HPPH which despite elevated levels, did not reach statistical significance (*p *> 0.05). In contrast, exposure of macrophages to 50 μg/mL of either PHT or HPPH prior to LPS stimulation prevented a significant increase in MMP-9 and TIMP-1 (Fig. 1D and Fig. 2). Levels of MMP-9 and TIMP-1 remained near control levels despite the potent proinflammatory challenge thus demonstrating the ability of these agents to alter macrophage function. Compared to LPS alone, pretreatment with 50 μg/mL PHT significantly blunted LPS-induced levels of MMP-1 (*p *< 0.05). In cultures pretreated with 50 μg/mL HPPH, MMP-3 levels were not significantly different compared to LPS-only treated cultures (*p *> 0.05) although the trend for reduced supernatant levels of MMP3 was evident. However, exposure of macrophages to either 15 μg/mL or 50 μg/mL PHT prior to LPS stimulation significantly blunted supernatant MMP-3 levels (*p *< 0.01 and *p *< 0.001, respectively, Fig. 1C) compared to LPS-only treated cultures. Interestingly, a trend for higher levels of MMP-1 were noted in cultures treated with HPPH while MMP-3 levels were slightly elevated in cultures treated with either PHT and HPPH although neither reached statistical significance (*p *> 0.05) (Fig. 1, A, C).

Elevated levels (50 μg/mL) of PHT or HPPH significantly reduced MMP-9 and TIMP-1 levels compared to LPS-only treated cells (Fig. 1D and Fig. 2). The levels of these analytes remained near control values despite LPS challenge. Interestingly, HPPH but not PHT was associated with reduced levels of MMP-2 compared to LPS only, but this relationship was not statistically significant. MMP-12 and TIMPs-2-4 remained below levels of detection in all groups and cultures.

### Supernatant levels of TNF-α but not IL-6, is decreased in response to PHT

**Figure 3:**
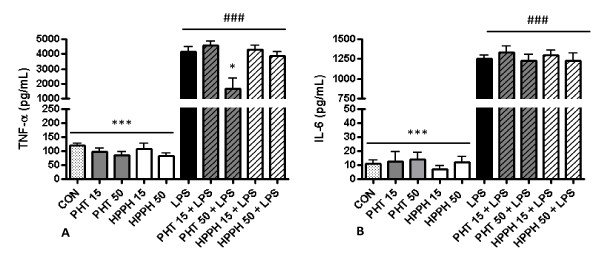
**The effect of phenytoin, HPPH and LPS on levels of (A) TNF-α and (B) IL-6 in conditioned medium from macrophage cultures**. Primary human monocyte-derived macrophages (*n *= 6 independent cultures) were pretreated with phenytoin or HPPH (15 μg/mL and 50 μg/mL) for 1 hour prior to challenge with 100 ng/mL *A. actinomycetemcomitans *LPS and the levels of TNF-α and IL-6 measured in conditioned media after 24 hours by enzyme-linked immunosorbent assay (ELISA). TNF-α, tumor necrosis-alpha; IL, interleukin; CON, control; PHT, phenytoin; HPPH, 5-(4-hydroxyphenyl-), 5-phenylhydantoin; LPS, lipopolysaccharide. Compared to CON, # *p *< 0.05, ## *p *< 0.01, ### *p *< 0.001, compared to LPS, * *p *< 0.05, ** *p *< 0.01, *** *p *< 0.001. Student *t*-test, *n *= 6 independent donors.

At 24 hours, supernatant levels of TNF-α and IL-6 were significantly increased by LPS compared to untreated controls (*p *< 0.001). Similar to MMP and TIMP levels, no significant differences in TNF-α and IL-6 levels were observed in supernatants exposed to either 15 or 50 μg/mL PHT and HPPH alone compared to untreated cultures although a trend for decreased levels of TNF-α was evident (Fig 3A). However, macrophage cultures pretreated with 50 μg/mL PHT prior to challenge with LPS showed a significant (*p *< 0.05) decrease in TNF-α levels compared to LPS only treated cultures. No difference was noted for 15 μg/mL of PHT or HPPH at either concentration (Fig. 3A). Regardless of dosage, pretreatment with PHT or HPPH prior to LPS challenge had no significant effect (*p *> 0.05) on IL-6 secretion when compared to LPS only treated cultures.

## Discussion

Macrophages are involved in a remarkably diverse array of homeostatic processes of vital importance to the host. In addition to their critical role in immunity [12], macrophages are also widely recognized as ubiquitous mediators of cellular turnover and maintenance of extracellular matrix homeostasis [13-18]. However, beyond their essentiality in immunity and tissue homeostasis, the macrophage has also been implicated in the evolution of periodontal pathological processes including periodontal disease and DIGO [11,19,20,31,32]. This investigation posited that macrophage-derived expression of proinflammatory cytokines, MMPs and/or TIMP expression is blunted upon exposure to PHT and/or HPPH hindering the ability of these cells to contribute to the fibroblast-mediated degradation of exuberant ECM proteins seen in DIGO. Since plaque-induced gingival inflammation exacerbates the manifestations of PHT-induced GO [33], we exposed macrophage cultures to purified LPS from the periodontal pathogen, *A. actinomycetemcomitans *(Aa) and examined protein levels of MMPs, TIMPs and proinflammatory cytokines in conditioned media. Aa can be isolated from plaque samples of patients with GO [34] while Aa LPS, a TLR4 agonist, strongly induces MMP and pro-inflammatory cytokine expression [28-30,35].

We exposed macrophage cultures to 2 different concentrations of PHT and HPPH. And while PHT plasma levels of 10-20 μg/mL are necessary to effectively maintain effective seizure control [22-24], disturbances in plasma as well as gingival concentrations of PHT are likely associated with DIGO. Indeed, Güncü *et al *[36] compared PHT levels in plasma and gingival crevicular fluid (GCF), a serum exudate, from subjects who demonstrated gingival overgrowth (responders) vs. those who did not (non-responders). Although PHT was detected in all of the GCF and plasma samples, the mean concentration of PHT was significantly greater in GCF compared to plasma (294.99 ± 430.15 μg/mL vs. 16.09 ± 4.21 μg/mL, respectively). Further, the concentration of plasma PHT was significantly higher in responders compared to non-responders (16.09 ± 4.21 μg/mL vs. 9.93 ± 4.56, respectively).

MMP-1 is recognized as an important mediator of connective tissue remodeling reported to be present at high concentrations in inflamed gingiva [37]. In the present study, supernatant MMP levels did not demonstrate any significant differences in response to PHT and HPPH alone at either dose compared to untreated macrophage cultures although we noted a trend for higher levels of MMP-1 and MMP-3. This finding was attributed to donor-specific variations in responses to these agents and serve to highlight clinical observations that approximately 50% of patients taking PHT develop GO [1,2]. This notion is supported by the finding that fibroblasts derived from subjects with cyclosporine-A (CSA)-induced gingival overgrowth produce significantly lower levels of MMP-1 than fibroblasts derived from subjects without overgrowth [38]. In the present study, supernatant levels of several MMPs were significantly decreased relative to LPS-only cultures in a dose-dependent manner suggesting that PHT and HPPH may mitigate the macrophage's ability to degrade ECM proteins by limiting its natural response to produce metalloproteinases. Such a dose response is consistent with other studies which have demonstrated, not only a similar effect on MMP-1 and MMP-3 at the protein and mRNA level [39-43], but also that a threshold of serum concentration of CSA helps to govern this mechanism [44-49].

MMP activity is counteracted by the actions of TIMPs. Here we report that exposure of macrophages to LPS was associated with an increase in TIMP-1 levels while exposure to high concentration (50 μg/mL) of PHT and HPPH, on the other hand, significantly reduced TIMP-1 levels. This finding is in agreement with *in-vitro *and *in-vivo *studies which report a relative reduction in MMP-1 and MMP-8/TIMP-1 in gingival fibroblasts and in serum and GCF concentration in CSA-associated gingival overgrowth subjects [50,51]. This reflects more a decrease in MMP production rather than an increase in TIMP. In fact, this corresponds with our findings in that supernatant levels of TIMP-1 in samples treated with both LPS and high doses of PHT or HPPH were not significantly different relative to untreated controls (Fig. 2). The net effect on ECM metabolism is based on the relative ratios of MMP and TIMP. When MMP levels decrease and/or TIMP levels increase, the turnover of ECM diminishes, potentially leading to an exuberant accumulation of these proteins. In this study, elevated levels of PHT in LPS-stimulated macrophages were associated with decreases in both MMP and TIMP levels. Therefore the decrease in TIMP-1 levels was counteracted by decreases in MMP levels. As a result, the macrophage's synergistic relationship with the fibroblast would be compromised leading to DIGO. Indeed, monocytes (macrophage precursors) can stimulate fibroblasts to produce MMP-1 by cell-cell interactions while conditioned media from monocytes is capable of inducing MMP-1 production in fibroblasts [52]. How PHT and HPPH impact monocyte/macrophage-fibroblast interactions and MMP production requires further study.

PHT is known to affect Na^+ ^as well as Ca^2+ ^metabolism [3], (e.g., Ca^2+ ^channels) and it is likely that this will impact MMP/TIMP and cytokine levels [53]. Indeed, Na^+ ^channels have been linked to activation of macrophages and microglia [54] and accumulating evidence indicates that sodium channel blockers can contribute to modulation of immune functions [55]. PHT has been reported to ameliorate the inflammatory response associated with experimental autoimmune encephalomyelitis in mice [54], modulate intracellular signaling cascades to TLR ligands [56] and significantly reduce LPS-induced phagocytosis *in-vitro*[53]. Here we report a dose-dependent inhibition of macrophage function by way of suppressed supernatant levels of MMP-1, MMP-3, MMP-9, TIMP-1 and TNF-α by PHT in human macrophages challenged with LPS. PHT has been reported to inhibit both activation of T-type calcium channels and RANKL-induced expression of c-*fos *protein in bone marrow-derived macrophages implying that calcium signals play a role in c-*fos *expression [57]. PHT was also shown to inhibit NFATc1 signaling in these cells. Further, in atrial myocytes, pharmacological inhibition of NFAT with 11R-VIVIT almost completely blunted the stretch-induced up-regulation of active-MMP-2/-9 [58]. Kiode *et al *[57] suggested that PHT may inhibit NFATc1 signals through suppression of c-*fos *expression. Since c-*fos*/AP-1 regulates the expression of numerous inflammatory cytokines and MMPs/TIMPs via promoter AP-1 binding motif [59,60], suppression of c-*fos *may provide a possible mechanism whereby MMPs/TIMPs and possibly cytokine levels are inhibited.

In contrast to PHT we report a dose-dependent inhibition of MMP-9 and TIMP-1 by HPPH in cultures challenged with LPS. These discrepancies may be attributed to differences in the interactions of these drugs with target molecules. Kobayashi *et al *[61] reported that PHT and 5-(4-methylphenyl)-5-phenylhydantoin, which contain a phenyl or methylphenyl group at both R2- and R3-positions activated the ligand binding domain of human pregane X receptor (hPXR), a member of the nuclear receptor family of ligand-activated transcriptional factors, whereas 5-(4-hydroxyphenyl)-5-phenylhydantoin did not. Alternatively, it is possible that higher concentrations of HPPH may be required to achieve results similar to that observed with PHT as evident by the trend for blunting of MMP-1 and MMP3 at higher doses of HPPH (Fig. 1A, C). Nevertheless, these findings serve to highlight the impact of PHT and HPPH, on the macrophage's ability to contribute to ECM turnover and underscore the importance of Na^+ ^and Ca^2+ ^channels in activated macrophages.

An interesting finding of our study was the suppression of TNF-α but not IL-6 by PHT. IL-6 enhances proliferation of fibroblasts and exerts a positive effect on collagen and glycosaminoglycan synthesis [62,63]. At high levels, TNF-α has been reported to inhibit collagen synthesis [64] and increase MMP synthesis in gingival fibroblasts [65-67], which contributes to gingival breakdown. Conversely, at low levels (< 10 ng/ml) TNF-α stimulates cellular proliferation, induces production of ECM and inhibits phagocytosis of collagen by gingival fibroblasts [68,69]. Since TNF-α enhances MMP-1 [70] and MMP-9 [71] expression, the blunting of TNF-α levels observed in the present study may have contributed to the decrease in supernatant levels of MMP-1 and MMP-9. In microglial cells, blockade of sodium channels with PHT significantly reduced LPS-induced secretion of IL-1α, IL-1β, and TNF-α, but not IL-6 or IL-10 suggesting that sodium channels participate in the process of cytokine release [53]. In agreement, we noted specific modulation of LPS-induced TNF-α but not IL-6 in the presence of high concentrations of PHT (50 μg/ml). Black *et al *[53] also demonstrated that tetrodotoxin, a sodium channel blocker, inhibited secretion of IL-1α, IL-1β, and TNF-α secretion but to a lesser degree than PHT, in spite of similar inhibitory actions on sodium channels. This difference was likely due to the effects on Ca^2+ ^metabolism by PHT. It was also interesting to note that HPPH had no effect on TNF-α levels. As discussed above this may be due to differences in the interactions of HPPH with target molecules or that higher dose of HPPH is required for inhibition of TNF-α.

In macrophages, increased TNF-α production in response to LPS challenge is associated with a transient increase in intracellular calcium [72,73] so that intracellular calcium may participate as a second messenger in TLR4-dependent signaling [72,74]. Insight into a possible mechanism linking intracellular calcium and cytokine levels was recently demonstrated using RAW macrophages [75]. Using a pharmacological approach, Yamashiro *et al *[75] examined the role of transient receptor potential vanilloin 4 (TRPV2), a calcium permeable channel, in LPS-induced calcium mobilization and induction of cytokines. They reported that LPS-induced IL-6 production was due at least in part by calcium mobilization solely from *intracellular *sources and partly by entry of extracellular calcium through TRPV2. Further, they reported that in addition to calcium mobilization through the IP_3_-receptor, TRPV2-mediated intracellular calcium mobilization involved NFκB-dependent TNF-α and IL-6 expression, while extracellular calcium entry is involved in NFκB-independent IL-6 production. Collectively, these findings may provide insights into how PHT and HPPH modulate cytokine and possibly MMP/TIMP levels. Future studies will be necessary to evaluate the impact of these agents on intracellular and extracellular calcium levels in macrophages prior to LPS challenge and their correlation to cytokine and MMP/TIMP production.

## Conclusions

Our results demonstrate that PHT as well as its metabolite, HPPH significantly blunt *A. actinomycetemcomitans *LPS-induced levels of MMP-1, MMP-3, MMP-9 and TIMP-1 in a dose-dependent manner and that a high concentration of PHT significantly decreases TNF-α but not IL-6 levels in the human macrophage. Given the presence of significant numbers of macrophages in gingival tissues and the correlation between the quality of plaque control and fibrosis, our data reveals a mechanism whereby both PHT and its metabolite, HPPH dysregulate macrophage function. Blunting of macrophage derived MMPs and TNF-α by these agents in response to stimuli may permit collagen accumulation without the counteracting production of MMPs by these cells.

## Competing interests

The authors declare that they have no competing interests.

## Authors' contributions

RS, NA and SN contributed to the concept and design of the study, and to the manuscript writing. SN, RS and AA performed isolated of monocytes and culture of macrophages. RS and AA performed the MMP, TIMP protein assays, cytokine assays, and viability assays. RS, NA and SN performed the data analysis. All authors read and approved the final manuscript.
